# Coronary collaterals and risk for restenosis after percutaneous coronary interventions: a meta-analysis

**DOI:** 10.1186/1741-7015-10-62

**Published:** 2012-06-21

**Authors:** Pascal Meier, Andreas Indermuehle, Bertram Pitt, Tobias Traupe, Stefano F de Marchi, Tom Crake, Guido Knapp, Alexandra J Lansky, Christian Seiler

**Affiliations:** 1 The Heart Hospital London, University College London Hospital Trust, London, UK; 2 St Thomas' Hospital, Department of Cardiology, King's College London, UK; 3 University of Michigan Medical Center, Department of Cardiology, Ann Arbor, MI, USA; 4 University Hospital Bern, Department of Cardiology, Bern, Switzerland; 5 University of Oslo, Department of Cardiology, Oslo, Norway; 6 TU University Dortmund, Department of Statistics, Dortmund, Germany; 7 Yale University Medical Center, Department of Cardiology, New Haven, CT, USA

**Keywords:** coronary collateral circulation, meta-analysis, restenosis, therapy failure

## Abstract

**Background:**

The benefit of the coronary collateral circulation (natural bypass network) on survival is well established. However, data derived from smaller studies indicates that coronary collaterals may increase the risk for restenosis after percutaneous coronary interventions. The purpose of this systematic review and meta-analysis of observational studies was to explore the impact of the collateral circulation on the risk for restenosis.

**Methods:**

We searched the MEDLINE, EMBASE and ISI Web of Science databases (2001 to 15 July 2011). Random effects models were used to calculate summary risk ratios (RR) for restenosis. The primary endpoint was angiographic restenosis > 50%.

**Results:**

A total of 7 studies enrolling 1,425 subjects were integrated in this analysis. On average across studies, the presence of a good collateralization was predictive for restenosis (risk ratio (RR) 1.40 (95% CI 1.09 to 1.80); *P *= 0.009). This risk ratio was consistent in the subgroup analyses where collateralization was assessed with intracoronary pressure measurements (RR 1.37 (95% CI 1.03 to 1.83); *P *= 0.038) versus visual assessment (RR 1.41 (95% CI 1.00 to 1.99); *P *= 0.049). For the subgroup of patients with stable coronary artery disease (CAD), the RR for restenosis with 'good collaterals' was 1.64 (95% CI 1.14 to 2.35) compared to 'poor collaterals' (*P *= 0.008). For patients with acute myocardial infarction, however, the RR for restenosis with 'good collateralization' was only 1.23 (95% CI 0.89 to 1.69); *P *= 0.212.

**Conclusions:**

The risk of restenosis after percutaneous coronary intervention (PCI) is increased in patients with good coronary collateralization. Assessment of the coronary collateral circulation before PCI may be useful for risk stratification and for the choice of antiproliferative measures (drug-eluting stent instead bare-metal stent, cilostazol).

## Background

Coronary collaterals are present in normal and diseased hearts. This coronary collateral circulation (CCC) has the potential to alleviate myocardial ischemia [1,2]. There is strong evidence that the CCC has a positive impact on survival [3,4]. However, some data suggested an increased risk for restenosis following percutaneous coronary intervention (PCI) in patients with good collateralization; however, the findings derive from small studies and have been rather inconsistent [5,6]. The purpose of this systematic review and meta-analysis was to integrate all available data in order to provide a clearer understanding of the impact of the coronary collateral on the risk for restenosis following PCI.

## Methods

The study was performed according to the Meta-analysis Of Observational Studies in Epidemiology (MOOSE) guidelines for meta-analyses of observational data (Additional file 1) [7]. Planning and study design was performed by two authors (CS, PM) including creation of an electronic database with variables of interest (Microsoft Excel; Microsoft, Redmond, WA, USA). Primary and secondary endpoints, variables of interest and search strategy (databases, sources for unpublished data) were defined in a strategy outline that can be obtained from the study authors on request.

### Search strategy

We searched the EMBASE, PubMed, MEDLINE, BIOS, International Pharmaceutical Abstracts database, and ISI Web of Science databases from 1980 to 15 July 2011. In addition, abstract lists and conference proceedings from the 2006 to 2010 scientific meetings of the American College of Cardiology, the European Society of Cardiology, the symposium on Transcatheter Cardiovascular Therapeutics, the American Heart Association, and the World Congress of Cardiology were searched. We also considered published review articles, editorials, and internet-based sources of information http://www.tctmd.com, http://www.theheart.org, http://www.europcronline.com, http://www.cardiosource.com, and http://www.crtonline.com to assess potential information on studies of interest. Reference lists of selected articles were reviewed for other potentially relevant citations. No language restriction was applied. Authors of selected studies were contacted to obtain further information if needed. All prospective studies reporting on an association between restenosis probability and coronary collateral circulation were included in this analysis. Retrospective case-control studies were not considered. We focused on prospective cohort studies because our objective was to define the value of collaterals as a marker for future restenosis. Furthermore, we excluded retrospective case-control studies because we think that matching of cases with controls could introduce critical bias; and in many cases, collaterals cannot be assessed accurately in retrospect.

The detailed search syntax used for the Medline database is shown in Additional file 2. The syntax for other databases was similar, but was adapted where necessary.

### Study selection

**Figure 1:**
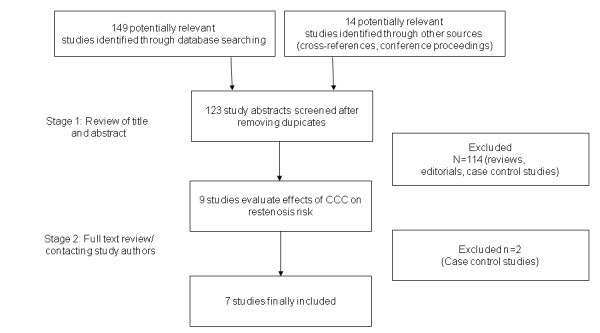
**Study selection process**.

In a two-step selection process, the titles and abstracts of all citations were reviewed by two researchers (PM, CS) to identify potentially relevant studies. In a second step, the full text of corresponding publications was reviewed to assess whether the studies met the following inclusion criteria: association of restenosis risk and the degree of coronary collateralization (Figure 1).

### Data extraction and quality assessment

Relevant information from the articles including baseline clinical characteristics of the study population and outcome measures was extracted using the prepared standardized extraction database (Excel). The quality of each study was assessed with the Newcastle-Ottawa Scale (NOS) for assessing the quality of non-randomized studies in meta-analyses (Table 1) [8]. Based on this scale, an additional sensitivity analysis of studies with superior quality was performed (at least seven of eight points). We did not use the quality scores for study weighting due to the limitations inherent to such an approach [9].

**Table 1:** Quality assessment of studies according to the Newcastle-Ottawa Scale

Lead author	Representativeness	Control group	Ascertainment	Endpoint not present at start	Assessment of outcome	Follow-up duration	Adequacy follow-up
Probst	*	*		*		*	*
Nakae	*	*		*		*	*
Wahl	*	*	*	*	*	*	
Perera	*	*	*	*	*	*	*
Jensen	*	*	*	*	*	*	*
Lee	*	*	*	*	*	*	*
Antoniucci	*	*		*		*	*

### Endpoints

The primary endpoint of this analysis was angiographic binary restenosis (> 50% restenosis). This dichotomized endpoint definition was selected because we expected that most studies report on dichotomized values; classically, a 50% cut off has been used in angioplasty studies. This value was mainly based on the experimental work of Gould *et al.*, demonstrating blunting of the hyperemic response at a stenosis degree of 50% [10].

### Definitions

Good collateralization was defined differently for the individual studies. Three studies performed a visual assessment (Rentrop score) [11] and used a score of ≤ 1 for poor collateralization (no or only faintly visible collaterals). In brief, the Rentrop score assigns four degrees of collateralization depending on the presence and extension of the collateral filling of coronary epicardial vessels during a coronary angiogram (grade 0 = no collaterals; grade 1 = side branch filling of the recipient artery without filling of the main epicardial artery; grade 2 = partial filling of the main epicardial recipient artery; grade 3 = complete filling of the main epicardial recipient artery). Four studies based their collateral quantification on intracoronary pressure measurements (collateral flow index (CFI)) [12] (Table 2) and defined poor collateralization as a CFI < 0.25. The CFI defines the proportion of blood flow that derives from the collateral circulation in comparison to the antegrade blood flow through the main coronary artery. The CFI is measured with a pressure sensor tipped coronary guidewire, which is placed in the distal vessel. The collateral flow index calculation is based on the occlusion pressure during a 1-minute balloon inflation and the pressure proximal to the balloon occlusion (aortic pressure). The central venous pressure is subtracted from these two pressures to correct for the back pressure: CFI = (occlusion pressure - central venous pressure) ÷ (systemic pressure - central venous pressure).

**Table 2:** Summary of the characteristics of the included studies

Lead author	Year	CCC assessment	Setting	Follow-up (months)	PCI type	Male (%)	Age (years)
Probst	1991	Visually	Elective	4 to 7	POBA 100%	79	51
Nakae	1996	Visually	Acute MI	Mean 5.7	POBA 100%	75	62
Wahl	2000	CFI	Elective	Mean 17	BMS 43%	74	60.5
Lee	2002	CFI	Acute MI	6	BMS 74.3%	73	57
Antoniucci	2002	Visually	Acute MI	6	BMS 64%	78	64
Perera	2006	CFI	Elective	6	BMS 100%	80	60
Jensen	2007	CFI	Elective	9	BMS 100%	75	61

BMS, bare metal stents; CCC, coronary collateral circulation; CFI, collateral flow index (intracoronary wedge-pressure derived collateral assessment); PCI, percutaneous coronary intervention; POBA, plain balloon angioplasty; Visually, collateral assessment with Rentrop scoring system.

### Data synthesis and analysis

Data from included studies were combined to estimate the pooled impact (risk ratio (RR)) of good collateralization versus poor collateralization. Calculations were based on a DerSirmonian and Laird random-effects model [13]. This model assumes that the true effects vary between studies for unknown reasons. The primary summary measure usually reported was the estimated average effect across studies [14]. Continuity correction was used when no event occurred in one group to allow calculation of a RR [15]. Heterogeneity among trials was quantified with the Higgins and Thompson I^2 ^[16]. I^2 ^can be interpreted as the percentage of variability due to heterogeneity between studies rather than sampling error. An I^2 ^> 50% was considerate as an at least moderate heterogeneity. We present as our primary results estimates of the average effect across studies with 95% confidence intervals in brackets. In addition, we also calculated 95% prediction intervals as described by Higgins *et al. *[14]. These intervals predict the effect that we would potentially expect to see in a new study. These data are presented in the Sensitivity analysis section. To assess the effect of moderator variables (study setting, method of collateral assessment, proportion of stent use, risk of restenosis in the control groups), a mixed-effects model was used (meta-regression). For binary moderator variables, we also present the ratio of risk ratios, which was calculated with the exponential function exp (estimated regression coefficient), with the according 95% confidence intervals. Prespecified subgroup analyses were 'setting' (elective PCI versus acute myocardial infarction (MI)) and 'collateral assessment method' (visual versus CFI). The remainder of the subgroup/meta-regression analyses were performed post hoc in an exploratory fashion.

To assess the effect of individual studies on the summary estimate of effect, we performed an influence analysis using a jackknife procedure; pooled estimates were recalculated by omitting one study at a time. We assessed publication bias visually (funnel plot) and by formal tests (rank order correlation test and Egger's test of intercept) [17,18]. All analyses were performed with R version 2.10.1 (package 'meta') [19].

## Results

### Description of included studies

A total of 123 articles were reviewed and 7 studies including 1,425 patients satisfied the predetermined inclusion criteria (Figure 1) [5,6,20-24]. The study of Wahl *et al. *has been published as a retrospective case-control study [25]. The study focused on patients with restenosis (case) and compared them to control patients without restenosis. The data used in this meta-analysis are based on a reanalysis of the identical cohort but with dividing patients into a group with 'good collateralization' and 'poor collateralization', depending on their CFI (< threshold versus ≥ 0.25), the incidence of restenosis (≥ 50% diameter stenosis) was calculated for the two groups (unpublished results). Table 2 summarizes the characteristics of the included studies. All patients had routine angiographic follow-up.

### Restenosis risk

**Figure 2:**
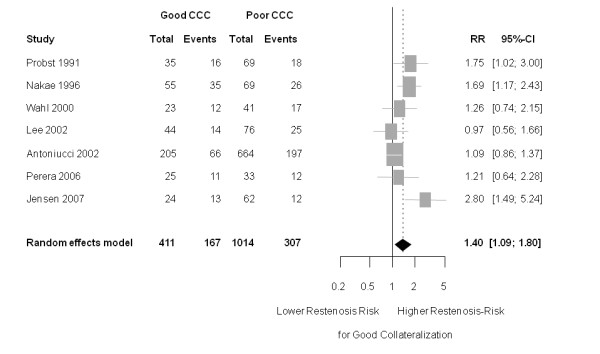
**Forest plot of risk ratios (RR) for restenosis (≥ 50% diameter stenosis)**. Markers represent point estimates of risk ratios, marker size represents study weight in random effects meta-analysis. Horizontal bars indicate 95% confidence intervals. CCC, coronary collateral circulation; CI, confidence interval.

Patients with a good collateralization showed a significantly increased risk for restenosis compared to patients with poor collateralization (RR 1.40 (95% CI 1.09 to 1.80); *P *= 0.009 (heterogeneity: tau^2 ^= 0.055; I^2 ^= 52.2% (95% CI 0% to 79.7%); *P *= 0.051) (Figure 2).

### Investigation of heterogeneity

**Figure 3:**
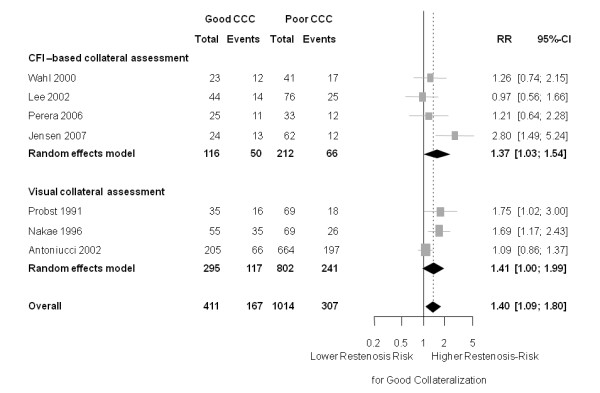
**Forest plot of risk ratios (RR) for restenosis (≥ 50% diameter stenosis), stratified by measurement method (CFI-based versus visual collateral assessment)**. Horizontal bars indicate 95% confidence intervals. CCC, coronary collateral circulation; CFI, collateral flow index; CI, confidence interval.

Analyses stratified by the method of collateral assessment showed very consistent results: good collaterals were associated with an increased restenosis risk. For visually assessed collateral assessment (based on Rentrop scoring) the RR was 1.41 ((95% CI 1.00 to 1.99); *P *= 0.049 (heterogeneity: tau^2 ^= 0.059; I^2 ^= 64.1%, *P *= 0.062)) and for CFI-based collateral assessment, the corresponding RR was 1.37 ((95% CI 1.03 to 1.83); *P *= 0.038 (heterogeneity: tau^2 ^= 0.112; I^2 ^= 56.1%, *P *= 0.077)) (Figure 3). There was no significant impact of the assessment method on the risk ratio in the according meta-regression analysis, the ratio of risk ratio between 'visual assessment' and 'CFI based assessment' was 1.02 (95% CI 0.59 to 1.71), *P *= 0.953.

The results were also very consistent between the two studies using plain balloon angioplasty (POBA) in all patients and those two studies using bare metal stents (BMS) (Table 2). The proportion of BMS used in the individual studies had no significant effect on the result neither (meta-regression: slope -0.15 (95% CI -10.83 to 0.54); *P *= 0.221; the meta-regression slope describes the impact of the proportion of BMS use on the study effect size; the log RR decreases on average by -0.15 when all the patients have a BMS implanted compared to the situation where none of the patients receives a BMS. (Additional file 3) Further, no significant effect of the restenosis risk in the control group (patients with poor collaterals) on the results was found neither (meta-regression: slope -2.44 (95% CI -6.54 to 1.66) *P *= 0.425); this means that the log RR decreases on average by -2.44 if the restenosis risk in the control group (poor collaterals) is 100% compared to the situation where the restenosis risk is 0% (Additional file 4).

**Figure 4:**
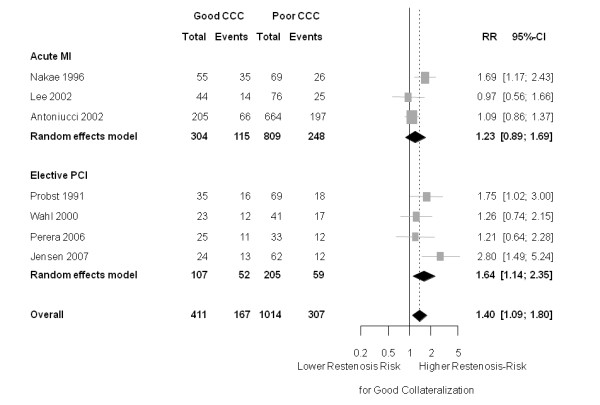
**Forest plot of risk ratios (RR) for restenosis (≥ 50% diameter stenosis), stratified by clinical setting (stable CAD versus acute MI)**. Horizontal bars indicate 95% confidence intervals. CAD, coronary artery disease; CCC, coronary collateral circulation; CFI, collateral flow index; CI, confidence interval; MI, myocardial infarction.

However, patients undergoing elective PCI for stable coronary artery disease (CAD) tended to show a more pronounced influence of collaterals on the restenosis risk. The risk ratio for those with good collateralization was 1.64 (95% CI 1.14 to 2.35); *P *= 0.008 (heterogeneity: tau^2 ^= 0.049; I^2 ^= 35.9%, *P *= 0.197)). For patients with acute myocardial infarction, however, the respective RR was 1.23 (95% CI 0.89 to 1.69); *P *= 0.212 (heterogeneity: tau^2 ^= 0.049; I^2 ^= 58.3%, *P *= 0.091)) (Figure 4). However, the according meta-regression analysis showed no statistically significant effect of this variable on the RR, the ratio of risk ratio between 'elective PCI' and 'acute MI' was 1.33 (95% CI 0.82 to 2.16), *P *= 0.243.

### Sensitivity analyses

**Figure 5:**
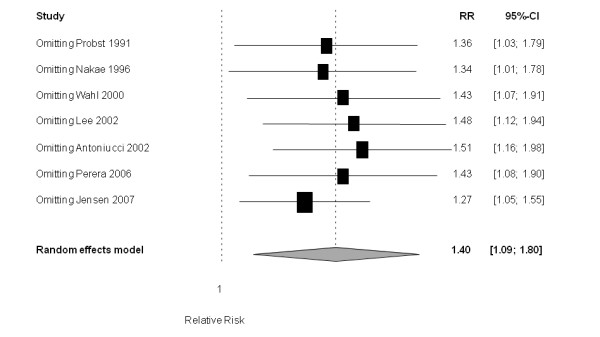
**Influence analysis with forest plot of risk ratios (RR) for restenosis**. Each line represents a reanalysis of the data with exclusion of one study (inclusion of six studies only) at a time to assess the influence of this particular study on the overall result.

None of the studies influenced the results to the extent that the conclusion would have changed; the jackknife procedure-based sensitivity analysis omitting one study at a time consistently showed that good collateralization is associated with an increased restenosis risk (Figure 5). Specifically, excluding the only unpublished data included in this analysis (based on Wahl *et al. *[25]) did not change the overall RR estimate (RR 1.43 (95% CI 1.07 to 1.91); *P *= 0.016 (heterogeneity: tau^2 ^= 0.074; I^2 ^= 60.1%, *P *= 0.028)). When considering studies with highest quality only (based on the Newcastle-Ottawa Scale; at least seven of eight points), the estimate for RR for restenosis for the group with good collaterals was very consistent but did not reach statistical significance (1.47 (95% CI 0.78 to 2.76); *P *= 0.235 (heterogeneity: tau^2 ^< 0.001; I^2 ^= 0%, *P *= 0.866)); three studies were considered in this analysis [5,6,24].

**Figure 6:**
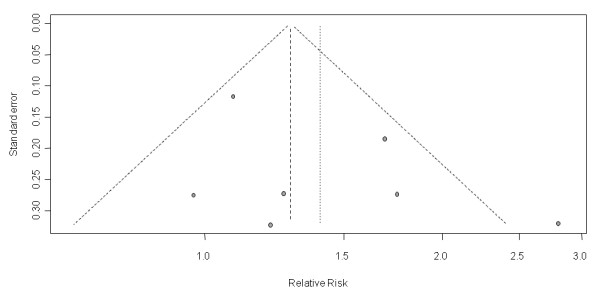
**Funnel plot of the estimates of relative risk versus standard error**. Lower standard errors indicate better precision and larger study size. SE, standard error.

The funnel plot was symmetrical (Figure 6) and formal testing did not indicate a relevant 'small study effect' or publication bias (Egger's test *P *= 0.362, rank correlation test *P *= 0.273).

Three studies enrolled patients with acute MI. While the visual assessment of collaterals should not be altered and may even be more accurate, because the vessel of interest is occluded and avoids 'competitive' flow via the native and the collateral vessels, the accuracy of quantitative CFI measurements have been questioned in this setting [26]. When excluding the one study using CFI measurements in acute MI [6], the overall result increases minimally, from RR 1.40 (95% CI 1.09 to 1.80) to RR 1.48 (95% CI 1.12 to 1.94); *P *= 0.005 (heterogeneity: tau^2 ^= 0.061; I^2 ^= 55.5%, *P *= 0.047). For the subgroup of acute MI patients, the RR increases from 1.23 (95% CI 0.89 to 1.69) to RR 1.32 (95% CI 0.86 to 2.04); *P *= 0.204 (heterogeneity: tau^2 ^= 0.075; I^2 ^= 75.7%, *P *= 0.043).

Lastly, we also calculated prediction intervals, which are wider compared to confidence intervals. For the overall results, including all studies, the RR was 1.40 with a prediction interval of 0.70 to 2.78. For the patients with elective PCI, it was 1.64 (0.47 to 5.65), for those with acute MI it was 1.23 (0.04 to 38.11).

This means that we predict that the true effect in a new study (assumed to be 'similar' to those studies included in the meta-analysis) would lie between RR = 0.70 and RR = 2.78 with 95% confidence. As such, although the average effect across studies of a 40% increase in restenosis in patients with a good collateralization is statistically significant, due to unexplained heterogeneity between existing results we cannot be sure of an effect in a new study.

## Discussion

This meta-analysis of seven studies shows that a 'good collateralization' is predictive for restenosis in patients undergoing PCI. This risk was found to be increased by 40% (95% CI 0% to 80%) compared to patients with poor collateralization. This association was found to be stronger in patients with stable coronary artery disease (risk increased by 64% (14% to 135%)) while it was weaker for patients with acute MI (risk increased by 23% (-11% to 69%)) and did not reach statistical significance in this subset. It has to be considered that the differences of the RR estimates between these subgroup analyses were not statistically significant. Moreover, all these values are estimates of the average effect across the different studies.

This data indicate that the degree of collateralization may be a useful and simple tool to inform individual clinical decision making, patients at high risk for restenosis may profit from the more expensive drug-eluting stents and from cilostazol, which both reduce the restenosis risk [27,28].

### Collaterals: good or bad?

Good coronary collateralization has been found to be associated with improved survival [3,4,29]. In this regard it may seem contradictory that good collateralization is a risk factor for restenosis after PCI. Similarly, accelerated disease progression of the native vessel after coronary artery bypass grafting (CABG) is a frequent phenomenon that does not affect the clinical benefit of CABG [30].

Coronary collaterals may be regarded as an analog to CABG in that both provide an alternative blood supply to the myocardium. Therefore, increased restenosis after PCI and improved survival benefit in patients with good collateralization are not mutually exclusive. Restenosis is usually a slow process and rarely results in a life-threatening event. This is demonstrated by the fact that most treatments that reduce the risk for restenosis, for example, drug eluting stents, do not result in improved survival.

### Potential mechanisms

One of the possible reasons for the increased risk of restenosis in case of a good collateralization is the flow via the collaterals, which competes with the antegrade blood flow through the native vessel [23,31,32]. As mentioned above, a similar phenomenon is frequently observed in native coronary arteries in proximity to a bypass graft, which also represents a collateral circulation, leading to decreased flow through the native vessel [31,32]. This reduced flow subsequently results in a decreased shear stress on the endothelial cell layer. This shear stress is known to be atheroprotective [33]. Monocytes and platelets are key players in the pathogenesis of intimal hyperplasia and atherosclerosis; low flow and low shear stress increases the chance of cell adhesion to the vessel walls [34]. Low shear stress also modulates endothelial cell gene expression into a proinflammatory state [35]. High shear stress, on the other hand, is suppressing the expression of these proinflammatory genes, specifically via the lung Kruppel-like factor (LKLF), an anti-inflammatory endothelial transcription factor [36,37]. LKLF also reduces the expression of the substance monocyte chemoattractant protein 1 (MCP-1). As its name suggests, MCP-1 attracts monocytes and has a proatherogenic effect [38]. However, the exact mechanism of this 'mechanotransduction', translating physical forces into changes at a molecular level, is not completely understood. Data suggest that G proteins may act as primary mechanosensors on endothelial cells; a further concept that has evolved are mechanosensitive ion channels that translate the physical force into a corresponding intracellular signal [39].

### Coronary collaterals as a marker or as a causal risk factor for restenosis?

The major determinant of collateral function is the degree of vessel stenosis, which itself has been described to increase restenosis risk [40-42]. The studies included in this meta-analysis did not adjust for covariates such as vessel diameter stenosis or the extent of CAD. As an alternative explanation, coronary collaterals could simply represent markers for more severe underlying CAD with consecutive increased risk for restenosis after PCI. This interesting question remains to be resolved in future studies. Regardless of a causal or a casual association, the degree of coronary collateralization represents a valuable and simple marker to predict the risk for restenosis.

### Outlook

Future research should evaluate possible mechanisms of this increased restenosis risk in patients with good collateralization. This patient group may show different levels of cytokine activation, inflammation, levels of reactive oxygen species (ROS) or platelet activation after percutaneous transluminal coronary angioplasty (PTCA), which may be addressed by additional pharmacologic approaches. One hypothesis to be tested is that the oxygen level distal to the vessel occlusion during angioplasty varies with varying collateralization and may lead to different ROS levels. Higher ROS levels may damage endothelial cells downstream and thereby increase the risk for restenosis.

### Limitations of this meta-analysis

Most studies used exclusively binary data for their analysis. The extent of variable of interest, collateralization, was dichotomized into 'good collateralization' and 'poor collateralization', while in fact the degree of collateralization is a continuous variable. Besides this variable of interest, the outcome was also dichotomized in most studies, using a restenosis threshold of 50%. One drawback of this approach is the impaired statistical power. Still, this meta-analysis was large enough to detect a significant influence of collaterals on restenosis risk. A related problem is the fact that all patients underwent routine angiographic follow-up. Some of the patients may have had a stable in-stent restenosis without symptoms, the routine angiographic follow-up may overrate the clinical importance of restenosis and it may overestimate the impact of collaterals on restenosis [43].

Further, the included studies did not adjust for potential confounding factors such as the severity of CAD, the diameter stenosis, and so on. Since this is a study-level and not a patient-level meta-analysis, we were not able to include these factors in our analyses. However, we think that significant confounding regarding our primary outcomes is rather unlikely. The main determinant of collaterals (our predictor) is the degree of the vessel diameter stenosis; the narrower the stenosis, the better the collaterals [40]. However, the degree of stenosis is not known to be a risk factor for future restenosis (primary outcome of our study).

Moreover, this study does not capture the dynamic of the coronary collaterals. The coronary collateral function has been demonstrated to decrease over a 6-month period after PCI [44]. This dynamic may explain the non-significant results in the setting of acute MI. During an acute vessel occlusion, the collaterals undergo rapid changes; a fact, that limits the value of a single timepoint measurement. Further, the increased left ventricular end diastolic pressure during acute MI impairs the accuracy of the collateral assessment [26].

Another important limitation is the heterogeneity among the studies included in this analysis. The extent of heterogeneity reduces the robustness of our results. We therefore performed several subset analyses and meta-regression analysis, and found several aspects that contribute to this heterogeneity. The most important one is the difference in study populations. Four studies included patients with stable CAD while three studies focused on patients with acute MI (Table 2). Further, the earliest two studies used plain balloon angioplasty while the newest studies used bare metal stents in all patients [5,24]. Also, four studies used CFI-based collateral assessment while three studies used visual assessment of collaterals. Despite this heterogeneity between studies, the findings were very consistent in most of the subset and sensitivity analyses. Moreover, the accuracy of CFI measurements in the setting of acute MI has been questioned [26]. However, this only applies to one study [6] and excluding this study only minimally influenced the overall results (see 'Sensitivity analyses').

A further drawback of this study is that early studies used plain balloon angioplasty and later studies used BMS. No drug-eluting stents (DES) were used in the present studies. Whether the results of this meta-analysis can be generalized to DES remains unanswered. DES have further reduced the risk for restenosis; it is highest for POBA (32% in average), around 22% for BMS and around 16% for first-generation DES [45,46]. The predictive value of collaterals may be reduced in the context of DES. However, even with DES, restenosis is still a significant and unresolved problem. Our findings were consistent in the POBA and in the BMS group; they were not significantly influenced by the proportion of BMS use in the individual studies or by the average restenosis risk in the control groups (poor collaterals). We would therefore expect similar results for drug-eluting stents.

Another limitation of our study is that it does not provide further insights into possible causal mechanisms of our findings. Our considerations in the Discussion are thus rather hypothetical. This study, overall, is hypothesis generating rather than confirmatory.

With regard to the meta-regression analyses, it has to be considered that they have limited statistical power and a lack of statistical significance does not necessarily mean that there is no true effect.

## Conclusions

The results of this meta-analysis including 1,425 patients show that a good coronary collateralization indicates an increased risk for restenosis. The degree of coronary collateralization may be useful information for clinical decision making during PCI, such as stent choice (DES versus BMS) and use of cilostazol, and it may also impact the aggressiveness of the post-PCI management.

## Competing interests

BP received consultant fees from Pfizer, Novartis, Merck, Takeda, Boehringer-Ingelheim, Bayer, Forrest Laboratories, GE Health Care, Relypsa, Nile Therapeutics, Aurasense and has stock options of Relypsa, Nile Therapeutics, Aurasense. BP received research grants from Medtronic, Novartis and Bayer. The other authors have no conflicts of interest to disclose.

## Authors' contributions

PM was responsible for the conception and design, acquisition of data, analysis and interpretation of data, drafting the initial manuscript and revising it critically for important intellectual content. AI was responsible for acquisition of data, data control, interpretation of data and for revising the manuscript critically for important intellectual content. TT, SFdM, TK, and AJL were responsible for revising the manuscript critically for important intellectual content. GK was responsible for analysis and interpretation of data and for revising the manuscript critically for important intellectual content. CS was responsible for the conception and design, acquisition of data, interpretation of data, and for revising the manuscript critically for important intellectual content. All authors read and approved the final manuscript.

## Author information

PM is an interventional Cardiologist at UCLH, London and is leading the Yale-UCL Device Development Program. He has a degree in applied statistics and has several years of experience in clinical research on the coronary collateral circulation and on meta-analyses (for more information, see http://www.drpascalmeier.com). BP is a Cardiologist and Professor at the University of Michigan. He was among the first researchers to demonstrate the existence of collateral circulation in the heart, published in 1959. GK is an Associate Professor for Statistics at TU University, Dortmund. He has developed meta-analysis methods, such as the Hartung-Knapp method, which is implemented in advanced statistics software and has coauthored a book on statistical aspects of meta-analyses. AJL is a cardiologist and an Associate Professor at Yale University and an expert in PCI research and restenosis; she has had a leading role in many of the landmark trials in this field. CS is an interventional cardiologist and Professor at University Hospital Bern, and has long-standing experience in clinical research on coronary collateral circulation.

## Pre-publication history

The pre-publication history for this paper can be accessed here:

http://www.biomedcentral.com/1741-7015/10/62/prepub

## Supplementary Material

Additional file 1 **MOOSE Checklist**. The MOOSE (Meta-analysis Of Observational Studies in Epidemiology) checklist is recommended to report on meta-analyses of observational studies and aims to ensure sufficient information about background, search strategy, methods, results, discussion, and conclusion. Click here for file

Additional file 2 **Search strategy**. Detailed description of search strategy and search terms used for the systematic review. Click here for file

Additional file 3 **Meta-regression of stent effect**. Meta-regression analysis of the proportion of bare-metal stents used versus the relative risk estimates. Click here for file

Additional file 4 **Meta-regression of restenosis risk effect**. Meta-regression analysis of the restenosis risk in the control group (poor collaterals) versus the relative risk estimates. Click here for file

## References

[B1] Seiler C The human coronary collateral circulation Eur J Clin Invest 40 465 476 20534067 10.1111/j.1365-2362.2010.02282.x

[B2] Pitt B Interarterial coronary anastomoses. Occurrence in normal hearts and in certain pathologic conditions Circulation 1959 20 816 822 10.1161/01.CIR.20.5.816 14433299

[B3] Meier P Gloekler S Zbinden R Beckh S de Marchi SF Zbinden S Wustmann K Billinger M Vogel R Cook S Wenaweser P Togni M Windecker S Meier B Seiler C Beneficial effect of recruitable collaterals: a 10-year follow-up study in patients with stable coronary artery disease undergoing quantitative collateral measurements Circulation 2007 116 975 983 10.1161/CIRCULATIONAHA.107.703959 17679611

[B4] Meier P Hemingway H Lansky AJ Knapp G Pitt B Seiler C The impact of the coronary collateral circulation on mortality: a meta-analysis Eur Heart J 2012 33 614 621 10.1093/eurheartj/ehr308 21969521

[B5] Jensen LO Thayssen P Lassen JF Hansen HS Kelbaek H Junker A Pedersen KE Hansen KN Krusell LR Botker HE Thuesen L Recruitable collateral blood flow index predicts coronary instent restenosis after percutaneous coronary intervention Eur Heart J 2007 28 1820 1826 10.1093/eurheartj/ehm067 17456484

[B6] Lee CW Hong MK Choi SW Kim JH Kim JJ Park SW Park SJ Influence of coronary collateral flow on restenosis following primary angioplasty for acute myocardial infarction Catheter Cardiovasc Interv 2002 55 477 481 10.1002/ccd.10097 11948894

[B7] Stroup DF Berlin JA Morton SC Olkin I Williamson GD Rennie D Moher D Becker BJ Sipe TA Thacker SB Meta-analysis of observational studies in epidemiology: a proposal for reporting. Meta-analysis Of Observational Studies in Epidemiology (MOOSE) group JAMA 2000 283 2008 2012 10.1001/jama.283.15.2008 10789670

[B8] Wells GA Brodsky LO O'Connell D Shea B Henry D Mayank S Tugwell P An evaluation of the Newcastle Ottawa scale: an assessment tool for evaluating the quality of non-randomized studies (abstract) XI International Cochrane Colloquium Book of Abstracts, O-63 2003 Presented at the XI Cochrane Colloquium, Barcelona 26

[B9] Juni P Witschi A Bloch R Egger M The hazards of scoring the quality of clinical trials for meta-analysis JAMA 1999 282 1054 1060 10.1001/jama.282.11.1054 10493204

[B10] Gould KL Lipscomb K Hamilton GW Physiologic basis for assessing critical coronary stenosis. Instantaneous flow response and regional distribution during coronary hyperemia as measures of coronary flow reserve Am J Cardiol 1974 33 87 94 10.1016/0002-9149(74)90743-7 4808557

[B11] Rentrop KP Cohen M Blanke H Phillips RA Changes in collateral channel filling immediately after controlled coronary artery occlusion by an angioplasty balloon in human subjects J Am Coll Cardiol 1985 5 587 592 10.1016/S0735-1097(85)80380-6 3156171

[B12] Seiler C Fleisch M Garachemani A Meier B Coronary collateral quantitation in patients with coronary artery disease using intravascular flow velocity or pressure measurements J Am Coll Cardiol 1998 32 1272 1279 10.1016/S0735-1097(98)00384-2 9809936

[B13] DerSimonian R Laird N Meta-analysis in clinical trials Control Clin Trials 1986 7 177 188 10.1016/0197-2456(86)90046-2 3802833

[B14] Higgins JP Thompson SG Spiegelhalter DJ A re-evaluation of random-effects meta-analysis J R Stat Soc Ser A Stat Soc 2009 172 137 159 10.1111/j.1467-985X.2008.00552.x PMC2667312 19381330

[B15] Sankey S Weissfeld L Fine M Kapoor W An assesment of the use of of the continuity correction for sparse data in metanalysis Commun Stat B-Simul 1996 25 1031 1056 10.1080/03610919608813357

[B16] Higgins JP Thompson SG Quantifying heterogeneity in a meta-analysis Stat Med 2002 21 1539 1558 10.1002/sim.1186 12111919

[B17] Begg C Mazumdar M Operating characteristics of a rank correlation test for publication bias Biometrics 1994 50 1088 1101 10.2307/2533446 7786990

[B18] Egger M Davey Smith G Schneider M Minder C Bias in meta-analysis detected by a simple, graphical test BMJ 1997 315 629 634 10.1136/bmj.315.7109.629 9310563 PMC2127453

[B19] R Development Core Team R: A language and environment for statistical computing http://www.R-project.org

[B20] Antoniucci D Valenti R Moschi G Migliorini A Trapani M Santoro GM Bolognese L Cerisano G Buonamici P Dovellini EV Relation between preintervention angiographic evidence of coronary collateral circulation and clinical and angiographic outcomes after primary angioplasty or stenting for acute myocardial infarction Am J Cardiol 2002 89 121 125 10.1016/S0002-9149(01)02186-5 11792328

[B21] Probst P Baumgartner C Gottsauner-Wolf M The influence of the presence of collaterals on restenoses after PTCA Clin Cardiol 1991 14 803 807 10.1002/clc.4960141006 1954688

[B22] Nakae I Fujita M Fudo T Iwase T Tanaka T Tamaki S Nohara R Sasayama S Relation between preexistent coronary collateral circulation and the incidence of restenosis after successful primary coronary angioplasty for acute myocardial infarction J Am Coll Cardiol 1996 27 1688 1692 10.1016/0735-1097(96)00043-5 8636554

[B23] Wahl A Billinger M Fleisch M Meier B Seiler C Quantitatively assessed coronary collaterals and restenosis following percutaneous revascularization Eur Heart J 1998 19 Suppl 389 10.1053/euhj.2000.2129 11052842

[B24] Perera D Postema P Rashid R Patel S Blows L Marber M Redwood S Does a well developed collateral circulation predispose to restenosis after percutaneous coronary intervention? An intravascular ultrasound study Heart 2006 92 763 767 16216859 10.1136/hrt.2005.067322 PMC1860667

[B25] Wahl A Billinger M Fleisch M Meier B Seiler C Quantitatively assessed coronary collateral circulation and restenosis following percutaneous revascularization Eur Heart J 2000 21 1776 1784 10.1053/euhj.2000.2129 11052842

[B26] de Marchi SF Oswald P Windecker S Meier B Seiler C Reciprocal relationship between left ventricular filling pressure and the recruitable human coronary collateral circulation Eur Heart J 2005 26 558 566 15618046 10.1093/eurheartj/ehi051

[B27] Tamhane U Meier P Chetcuti S Chen KY Rha SW Grossman MP Gurm H Efficacy of cilostazol in reducing restenosis in patients undergoing contemporary stent based PCI: a meta-analysis of randomised controlled trials EuroIntervention 2009 5 384 393 10.4244/V5I3A60 19736165

[B28] Stettler C Allemann S Wandel S Kastrati A Morice MC Schömig A Pfisterer ME Stone GW Leon MB de Lezo JS Goy JJ Park SJ Sabaté M Suttorp MJ Kelbaek H Spaulding C Menichelli M Vermeersch P Dirksen MT Cervinka P De Carlo M Erglis A Chechi T Ortolani P Schalij MJ Diem P Meier B Windecker S Jüni P Drug eluting and bare metal stents in people with and without diabetes: collaborative network meta-analysis BMJ 2008 337 a1331 10.1136/bmj.a1331 18757996 PMC2527175

[B29] Meier P Gloekler S de Marchi SF Zbinden R Delacretaz E Seiler C An indicator of sudden cardiac death during brief coronary occlusion: electrocardiogram QT time and the role of collaterals Eur Heart J 2010 31 1197 1204 10.1093/eurheartj/ehp576 20038512

[B30] Hlatky MA Boothroyd DB Bravata DM Boersma E Booth J Brooks MM Carrié D Clayton TC Danchin N Flather M Hamm CW Hueb WA Kähler J Kelsey SF King SB Kosinski AS Lopes N McDonald KM Rodriguez A Serruys P Sigwart U Stables RH Owens DK Pocock SJ Coronary artery bypass surgery compared with percutaneous coronary interventions for multivessel disease: a collaborative analysis of individual patient data from ten randomised trials Lancet 2009 373 1190 1197 10.1016/S0140-6736(09)60552-3 19303634

[B31] Aldridge HE Trimble AS Progression of proximal coronary artery lesions to total occlusion after aorta-coronary saphenous vein bypass grafting J Thorac Cardiovasc Surg 1971 62 7 11 5091689

[B32] Cashin WL Sanmarco ME Nessim SA Blankenhorn DH Accelerated progression of atherosclerosis in coronary vessels with minimal lesions that are bypassed N Engl J Med 1984 311 824 828 10.1056/NEJM198409273111304 6332274

[B33] Stone PH Coskun AU Kinlay S Clark ME Sonka M Wahle A Ilegbusi OJ Yeghiazarians Y Popma JJ Orav J Kuntz RE Feldman CL Effect of endothelial shear stress on the progression of coronary artery disease, vascular remodeling, and in-stent restenosis in humans: in vivo 6-month follow-up study Circulation 2003 108 438 444 10.1161/01.CIR.0000080882.35274.AD 12860915

[B34] Fan L Karino T Effect of a disturbed flow on proliferation of the cells of a hybrid vascular graft Biorheology 47 31 38 20448296 10.3233/BIR-2010-0561

[B35] Pan S Molecular mechanisms responsible for the atheroprotective effects of laminar shear stress Antioxid Redox Signal 2009 11 1669 1682 10.1089/ars.2009.2487 19309258 PMC2842586

[B36] Dekker RJ van Soest S Fontijn RD Salamanca S de Groot PG VanBavel E Pannekoek H Horrevoets AJ Prolonged fluid shear stress induces a distinct set of endothelial cell genes, most specifically lung Kruppel-like factor (KLF2) Blood 2002 100 1689 1698 10.1182/blood-2002-01-0046 12176889

[B37] Parmar KM Larman HB Dai G Zhang Y Wang ET Moorthy SN Kratz JR Lin Z Jain MK Gimbrone MA Jr García-Cardeña G Integration of flow-dependent endothelial phenotypes by Kruppel-like factor 2 J Clin Invest 2006 116 49 58 16341264 10.1172/JCI24787 PMC1307560

[B38] Dekker RJ van Thienen JV Rohlena J de Jager SC Elderkamp YW Seppen J de Vries CJ Biessen EA van Berkel TJ Pannekoek H Horrevoets AJ Endothelial KLF2 links local arterial shear stress levels to the expression of vascular tone-regulating genes Am J Pathol 2005 167 609 618 10.1016/S0002-9440(10)63002-7 16049344 PMC1603569

[B39] Traub O Berk BC Laminar shear stress: mechanisms by which endothelial cells transduce an atheroprotective force Arterioscler Thromb Vasc Biol 1998 18 677 685 10.1161/01.ATV.18.5.677 9598824

[B40] Pohl T Seiler C Billinger M Herren E Wustmann K Mehta H Windecker S Eberli FR Meier B Frequency distribution of collateral flow and factors influencing collateral channel development. Functional collateral channel measurement in 450 patients with coronary artery disease J Am Coll Cardiol 2001 38 1872 1878 10.1016/S0735-1097(01)01675-8 11738287

[B41] Schaper W Collateral circulation: past and present Basic Res Cardiol 2009 104 5 21 10.1007/s00395-008-0760-x 19101749 PMC2755790

[B42] Wen S Mao J Guo L Multivariate analysis of clinical factors in restenosis after coronary interventional treatment [in Chinese] Zhonghua Yi Xue Za Zhi 1999 79 197 199 11601040

[B43] Pinto DS Stone GW Ellis SG Cox DA Hermiller J O'Shaughnessy C Mann JT Mehran R Na Y Turco M Caputo R Popma JJ Cutlip DE Russell ME Cohen DJ TAXUS-IV Investigators Impact of routine angiographic follow-up on the clinical benefits of paclitaxel-eluting stents: results from the TAXUS-IV trial J Am Coll Cardiol 2006 48 32 36 10.1016/j.jacc.2006.02.060 16814645

[B44] Perera D Kanaganayagam GS Saha M Rashid R Marber MS Redwood SR Coronary collaterals remain recruitable after percutaneous intervention Circulation 2007 115 2015 2021 10.1161/CIRCULATIONAHA.106.665257 17404157

[B45] Farooq V Gogas BD Serruys PW Restenosis: delineating the numerous causes of drug-eluting stent restenosis Circ Cardiovasc Interv 4 195 205 21505166 10.1161/CIRCINTERVENTIONS.110.959882

[B46] Serruys PW de Jaegere P Kiemeneij F Macaya C Rutsch W Heyndrickx G Emanuelsson H Marco J Legrand V Materne P Belardi J Sigwart U Colombo A Goy JJ van den Heuvel P Delcan J Morel M-a Benestent Study Group A comparison of balloon-expandable-stent implantation with balloon angioplasty in patients with coronary artery disease. Benestent Study Group N Engl J Med 1994 331 489 495 10.1056/NEJM199408253310801 8041413

