# Supplementary material for: Coronary collaterals and risk for restenosis after percutaneous coronary interventions: a meta-analysis
Source: BMC Med. 2012 Jun 21;10:62. doi: 10.1186/1741-7015-10-62 (PMC3386894; doi:10.1186/1741-7015-10-62)
Supplement: Additional file 2 — Search strategy. Detailed description of search strategy and search terms used for the systematic review. [file 1741-7015-10-62-S2.DOC]

| Additional File 2: Search strategy for MEDLINE (search date July 15, 2011). | |
| --- | --- |
| **Search** | **# of abstracts** |

Search Queries

#11 Search #6 AND #8 OR #9 76

#10 Search #5 AND #2 25

#9 Search "Treatment Failure""[All Fields] 27383

#8 Search "Restenosis"[All Fields] 16008

#6 Search "Coronary Collateral"[All Fields] 834

#5 Search #3 OR #4 26601

#4 Search "Treatment Failure"[Mesh] 24604

#3 Search "Coronary Restenosis"[Mesh] 4654

#2 Search "Collateral Circulation"[Mesh] 9583
