# Supplementary figures and images for: Coronary collaterals and risk for restenosis after percutaneous coronary interventions: a meta-analysis
Source: BMC Med. 2012 Jun 21;10:62. doi: 10.1186/1741-7015-10-62 (PMC3386894; doi:10.1186/1741-7015-10-62)

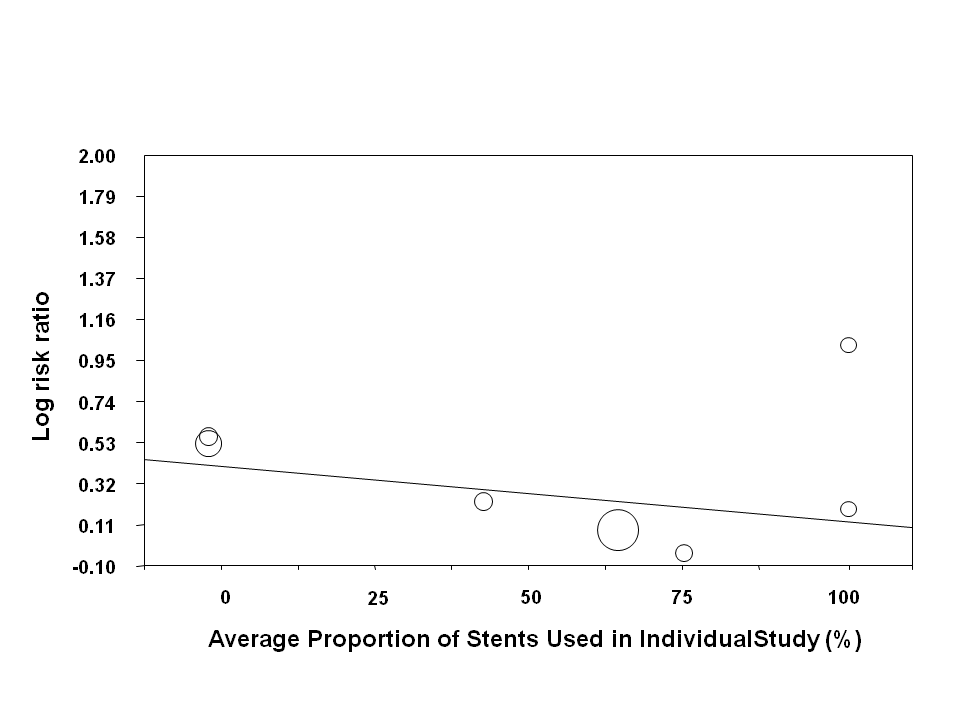

Supplement: Additional file 3 — Meta-regression of stent effect. Meta-regression analysis of the proportion of bare-metal stents used versus the relative risk estimates. [file 1741-7015-10-62-S3.TIFF]

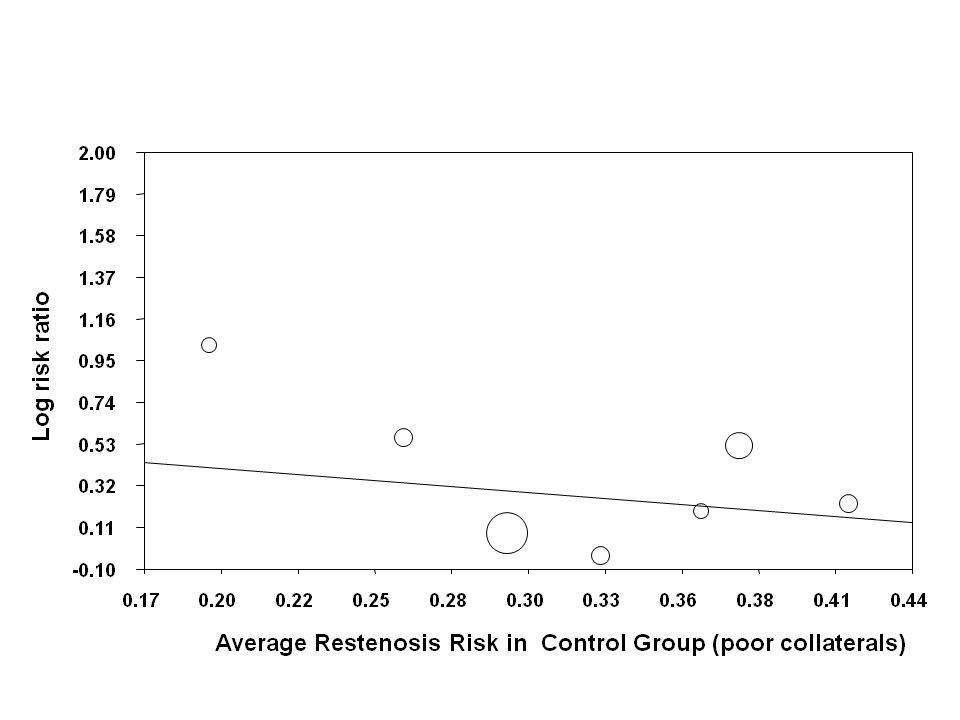

Supplement: Additional file 4 — Meta-regression of restenosis risk effect. Meta-regression analysis of the restenosis risk in the control group (poor collaterals) versus the relative risk estimates. [file 1741-7015-10-62-S4.TIFF]
